# Dietary Flaxseed Oil Prevents Western-Type Diet-Induced Nonalcoholic Fatty Liver Disease in Apolipoprotein-E Knockout Mice

**DOI:** 10.1155/2017/3256241

**Published:** 2017-09-07

**Authors:** Hao Han, Fubin Qiu, Haifeng Zhao, Haiying Tang, Xiuhua Li, Dongxing Shi

**Affiliations:** Department of Nutrition and Food Hygiene, School of Public Health, Shanxi Medical University, No. 56, Xinjian South Road, Taiyuan, Shanxi 030001, China

## Abstract

The prevalence of nonalcoholic fatty liver disease (NAFLD) has dramatically increased globally during recent decades. Intake of n-3 polyunsaturated fatty acids (PUFAs), mainly eicosapentaenoic acid (EPA, C20:5n-3) and docosahexaenoic acid (DHA, C22:6n-3), is believed to be beneficial to the development of NAFLD. However, little information is available with regard to the effect of flaxseed oil rich in *α*-linolenic acid (ALA, C18:3n-3), a plant-derived n-3 PUFA, in improving NAFLD. This study was to gain the effect of flaxseed oil on NAFLD and further investigate the underlying mechanisms. Apolipoprotein-E knockout (apoE-KO) mice were given a normal chow diet, a western-type high-fat and high-cholesterol diet (WTD), or a WTD diet containing 10% flaxseed oil (WTD + FO) for 12 weeks. Our data showed that consumption of flaxseed oil significantly improved WTD-induced NAFLD, as well as ameliorated impaired lipid homeostasis, attenuated oxidative stress, and inhibited inflammation. These data were associated with the modification effects on expression levels of genes involved in de novo fat synthesis (SREBP-1c, ACC), triacylglycerol catabolism (PPAR*α*, CPT1A, and ACOX1), inflammation (NF-*κ*B, IL-6, TNF-*α*, and MCP-1), and oxidative stress (ROS, MDA, GSH, and SOD).

## 1. Introduction

Nonalcoholic fatty liver disease (NAFLD) is the leading cause of chronic liver disease worldwide that is characterized by hepatocyte triacylglycerol accumulation (steatosis), which can progress to nonalcoholic steatohepatitis (NASH) or cirrhosis [1]. During recent decades, the prevalence of NAFLD has dramatically increased globally [2–4]. In addition, NAFLD has emerged not only as a liver disease causing considerable liver-related morbidity and mortality but also as a multisystem disease that affects many extrahepatic organ systems, including the heart and the vascular system [5]. However, at present, there are no approved pharmacological therapies for NAFLD in the clinic [6]. Therapeutic lifestyle interventions, typically focused on nutrition and exercise, are the keys in NAFLD treatment [7, 8]. Recent research has highlighted that decreasing the intake of total fats and increasing the intake of n-3 polyunsaturated fatty acids (PUFAs) might be beneficial in the treatment of NAFLD [9]. Delarue and Lallès gathered experimental and clinical data on the capability of long-chain n-3 PUFAs and found that eicosapentaenoic acid (EPA, C20:5n-3) and docosahexaenoic acid (DHA, C22:6n-3) supplement prevented or alleviated NAFLD [10]. The latest meta-analysis review showed beneficial changes in liver fat favored n-3 PUFA in adult NAFLD patients [11]. Moreover, a recent study presented by Yuan et al. demonstrated that fish oil rich in DHA and EPA improved lipid metabolism in western-style diet-induced NALFD and possessed anti-inflammatory effects [12]. Although several reports have described the preventive effects of fish oil or EPA and DHA, the n-3 PUFAs derived principally from fish oil, in experimentally induced NAFLD, there is a lack of evidence regarding the effects on NAFLD with dietary supplementation of flaxseed oil.

Flaxseed oil is a particularly rich source of *α*-linolenic acid (ALA, C18:3n-3), a plant-derived n-3 PUFA. Recently, flaxseed oil has been reported to ameliorate impaired lipid homeostasis with concurrent modification of proinflammatory mediators and oxidative stress, which play a key role in the development of NAFLD [13–15]. Thus, it is possible that supplementation of flaxseed oil could attenuate NAFLD. However, the impact of flaxseed oil on NAFLD has not been addressed.

The purpose of the present study was to determine the effects of dietary supplementation of flaxseed oil on NAFLD induced by western-type high-fat and high-cholesterol diet (WTD) in apolipoprotein-E knockout (apoE-KO) mice and investigate the underlying molecular mechanisms.

## 2. Materials and Methods

### 2.1. Chemical Sources

Commercial deodorized lard (food grade) was purchased from a local supermarket. The flaxseed oil (food grade) was manufactured by Caoyuankangshen Food Co. Ltd (Inner Mongolia, China). The fatty acid compositions of these experimental oils are shown in Table 1.

### 2.2. Animals

Male apoE-KO mice (4 weeks), purchased from Peking University Resources Centre (Beijing, China) with body weight of 15–19 g, were cared for according to the *Guide for the Care and Use of Laboratory Animals* published by the US National Institutes of Health.

### 2.3. Experimental Procedures

The mice were acclimatized to laboratory conditions for 1 week before the experiment and randomly allocated to three groups of 12 animals each. Each group was fed one of the following three diets for 12 weeks: normal chow diet with 20.7 kcal% fat (control group), western-style lard-rich diet with 41.5% kcal fat and 1.5% cholesterol (*w*/*w*) (WTD group) [16], or flaxseed-oil-rich western-style diet with a total 41.5% kcal fat containing 10% flaxseed oil (*w*/*w*) and with 1.5% cholesterol (*w*/*w*) (WTD + FO group) (Table 2).

After 12 weeks, the animals were fasted for 12 h and sacrificed. Blood samples were rapidly obtained by cardiac puncture. Serum was prepared from blood centrifuged at 3000*g* for 10 min at 4°C. Liver tissues were frozen immediately in liquid nitrogen and stored at −80°C or fixed in 4% buffered formalin.

### 2.4. Histology and Morphometry Evaluations of Lipid Deposition in Liver Tissue

Lipid deposition in liver tissue was evaluated according to the method of our previous study [17]. Fresh samples from the same position of the liver were divided into two parts. One part of samples were fixed in 4% paraformaldehyde and embedded in paraffin. Cross sections (5 *μ*m thick) obtained from the paraffin blocks were stained with hematoxylin and eosin (H&E). The other part of samples was embedded in tissue freezing OCT medium and sectioned into consecutive 5 *μ*m thick sections. Every sixth section was stained with Oil Red O and digitally photographed under ×200 magnification.

### 2.5. Measurement of Lipid Parameters in Serum and Liver

The concentrations of total cholesterol (TC), triglyceride (TG), low-density lipoprotein cholesterol (LDL-C), and high-density lipoprotein cholesterol (HDL-C) in serum and liver were measured by enzymatic colorimetric assays using commercially available detection kits (BioSino Bio-Technology Co. Ltd, Beijing, China).

### 2.6. Detection of Reactive Oxygen Species (ROS) Levels in Liver of Mice

Fresh cross sections (5 *μ*m) of unfixed but frozen liver were immediately incubated with 5 M dihydroethidium at 37°C for 15 min in a humidified chamber. Then, fluorescence level was visualized with a fluorescence microscope. Fluorescence intensities of the images were quantified by using the Image-Pro Plus image analysis.

### 2.7. Determination of Oxidative Stress Parameters in Serum and Liver

The concentration of malondialdehyde (MDA), an index of lipid peroxidation, was measured by using thiobarbituric acid colorimetry slightly modified by Ohkawa et al. [18]. The level of glutathione (GSH) was measured according to its reaction with 5,5′-dithiobis-2-nitrobenzoic acid (DTNB) into 2-nitro-5-thiobenzoic acid (TNB), following deproteinization by 5% trichloroacetic acid [19]. The activities of superoxide dismutase (SOD) in serum and liver were measured with enzymatic colorimetric assays according to the method of Kono [20]. The contents of MDA, GSH, and SOD in liver were standardized by protein concentration measured by the Bio-Rad Protein Assay Kit (USA).

### 2.8. Determination of Serum Biomarkers for Liver Injury

Serum aspartate aminotransferases (AST) and alanine aminotransferases (ALT) were measured with an enzymatic kinetic method by Mindray BS-200 automatic biochemistry analyzer (Shenzhen, China) with matching kits. Results were expressed as units per liter (U/L).

### 2.9. Evaluation of Inflammation Cytokines in Plasma

The levels of interleukin 6 (IL-6), tumor necrosis factor alpha (TNF-*α*), and monocyte chemotactic protein 1 (MCP-1) in plasma were measured by enzyme-linked immunosorbent assays according to the manufacturer's instructions of commercially available detection kits (R&D Systems, USA).

### 2.10. Real-Time RT-PCR Analysis

Total RNA was isolated from the stored frozen liver using the TRIzol reagent. (Invitrogen, 154 Carlsbad, CA, USA). Messenger RNA (mRNA) expression was quantified by using specific oligo primers and SYBR green-based qRT-PCR kit (TaKaRa Biotechnology Co. Ltd, Dalian, China) in the 7900HT instrument (Applied Biosystems, Foster, CA, USA). The specificity of the product was assessed from melting curve analysis. Gene expressions were determined using the 2^−ΔΔCt^ method. The mRNA of *β*-actin was quantified as an endogenous control. Gene expressions are presented as fold change relative to control. Quantitative real-time RT-PCR primers are shown in Table 3.

### 2.11. Western Blot Analysis

Liver tissues were homogenized and lysed in RIPA Lysis Buffer (1% Triton X-100, 1% deoxycholate, and 0.1% SDS). Total protein was determined according to previously described [21]. Tissue lysates with equal protein amounts were subjected to Western blotting. The protein was separated by 10% SDS-polyacrylamide gel and then transferred onto a PVDF membrane. The membranes were incubated with specific primary antibodies overnight at under 4°C after blocking with 5% nonfat milk solution. Then, the target proteins were incubated with the species-specific second antibodies conjugated to horseradish peroxidase. Immunoreactive bands were detected by means of an ECL plus Western Blotting Detection System (Amersham BioSciences, Little Chalfont, UK) according to the manufacturer's instructions. Quantitative analysis of the relative density of the bands in Western blots was performed by Quantity One 4.62 software (Bio-Rad, Hercules, CA, USA). Data were corrected for background standardized to *β*-actin as optical density (OD/mm^2^). Primary antibodies were as follows: monoclonal anti-*β*-actin antibody (Sigma), rabbit anti-PPAR*α* antibody (Abcam Limited), rabbit anti-CPT1A antibody (Sigma), rabbit anti-ACOX1 antibody (Abcam Limited), rabbit anti-SREBP-1c antibody (Abcam Limited), mouse anti-ACC antibody (R&D systems), rabbit antibodies against phosphorylated-p65 and p65 (Cell Signaling Technology), rabbit anti-IL-6 antibody (Cell Signaling Technology), mouse anti-TNF alpha antibody (Abcam Limited), rabbit anti-MCP-1 antibody (Abcam Limited), anti-rabbit IgG HRP-linked antibody (Cell Signaling Technology), and anti-mouse IgG HRP-linked antibody (Cell Signaling Technology).

### 2.12. Statistical Analysis

Results are expressed as means ± SEM, and *P* < 0.05 was considered significant. Statistical analyses of data were performed using one-way analysis of variance with SPSS 12.0 software package (SN: 59245 46841 40655 89389 09859 21671 21957 29589 12).

## 3. Results

### 3.1. Effects of Flaxseed Oil on Body and Liver Weight

Treatment of flaxseed oil had no effects on body weight change compared with WTD-fed mice but significantly decreased liver weight increased by WTD (Table 4).

### 3.2. Effects of Flaxseed Oil on Liver Morphology

**Figure 1:**
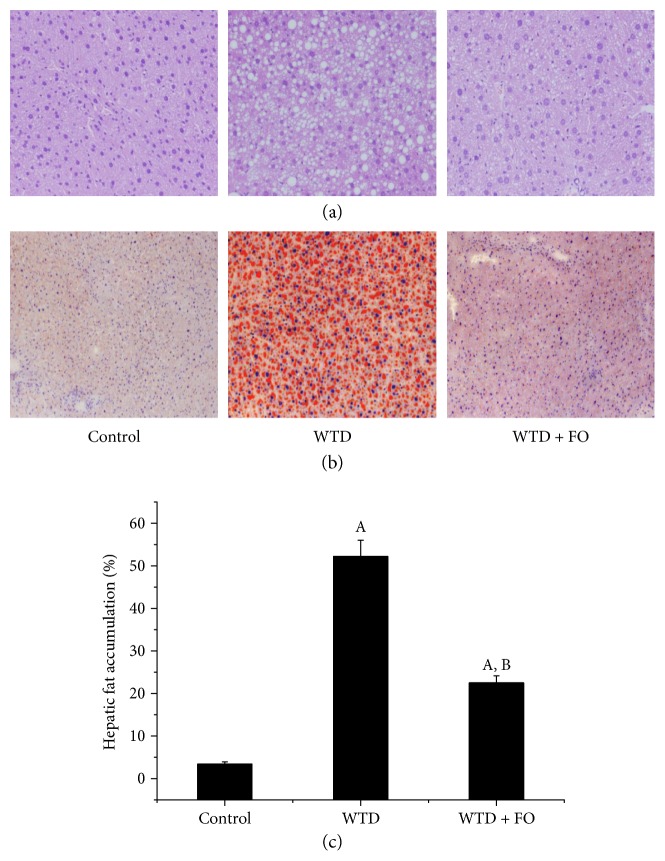
Dietary flaxseed oil ameliorated hepatic steatosis in mice. (a) H&E and (b) Oil Red O staining of lipid droplets in the livers of mice in each group (magnification ×200). (c) Quantitative analysis of hepatic fat accumulation. Data represent means ± SEM and are normalized to % of field area (*n* = 6 mice). ^A^*P* < 0.05 versus the control group; ^B^*P* < 0.05 versus the WTD group.

H&E and Oil Red O staining of lipid deposition in liver were analyzed to evaluate the effect of flaxseed oil on hepatic steatosis. As representative results shown in Figures 1(a) and 1(b), the mice given WTD were characterized by a large number of macrovesicular steatosis, while the circular lipid droplet was markedly reduced in the liver of mice fed with flaxseed oil. Quantitative analysis of the lipid droplet in the liver of mice (Figure 1(c)) demonstrated statistically smaller lipid droplet size in FO-treated animals than in WTD-treated mice.

### 3.3. Dietary Flaxseed Oil Improved Hepatic Lipid Metabolism in Mice

**Figure 2:**
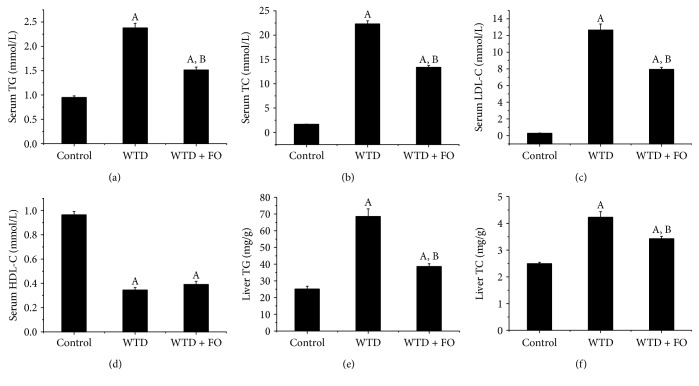
Flaxseed oil treatment improved lipid profiles in mice. (a) Serum TG, (b) serum TC, (c) serum LDL-C, (d) serum HDL-C, (e) liver TG, and (f) liver TC. Each bar or point denotes mean ± SEM (*n* = 12 mice). ^A^*P* < 0.05 versus the control group; ^B^*P* < 0.05 versus the WTD group.

As shown in Figure 2, high levels of TG, TC, and LDL-C in serum and liver induced by WTD were significantly reduced after exposure to flaxseed oil. However, flaxseed oil intervention had no effect on serum HDL-C.

**Figure 3:**
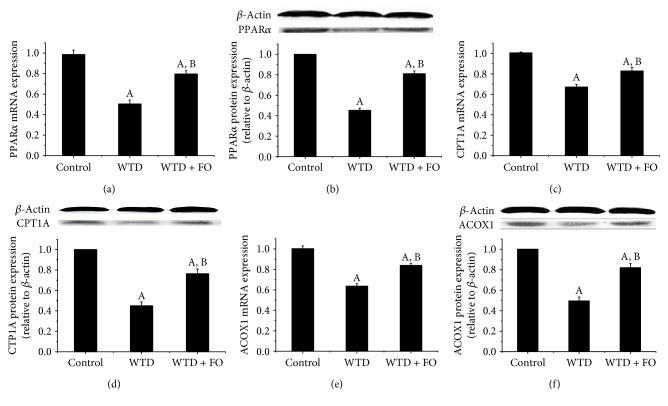
Flaxseed oil supplement increased mRNA and protein expressions of hepatic PPAR*α*, CPT1A, and ACOX1 in mice. Quantitative real-time RT-PCR and Western blot analysis of the mRNA and protein expressions of (a-b) PPAR*α*, (c-d) CPT1A, and (e-f) ACOX1 in the liver of mice in each group. Fold changes of mRNA levels were determined after normalization to internal control *β*-actin RNA levels. Blotting with anti-*β*-actin was used as a protein loading control. Protein expressions were presented as fold change relative to control. Representative immunoblots are shown. Each bar denotes mean ± SEM (*n* = 4 mice). ^A^*P* < 0.05 versus the control group; ^B^*P* < 0.05 versus the WTD group.

**Figure 4:**
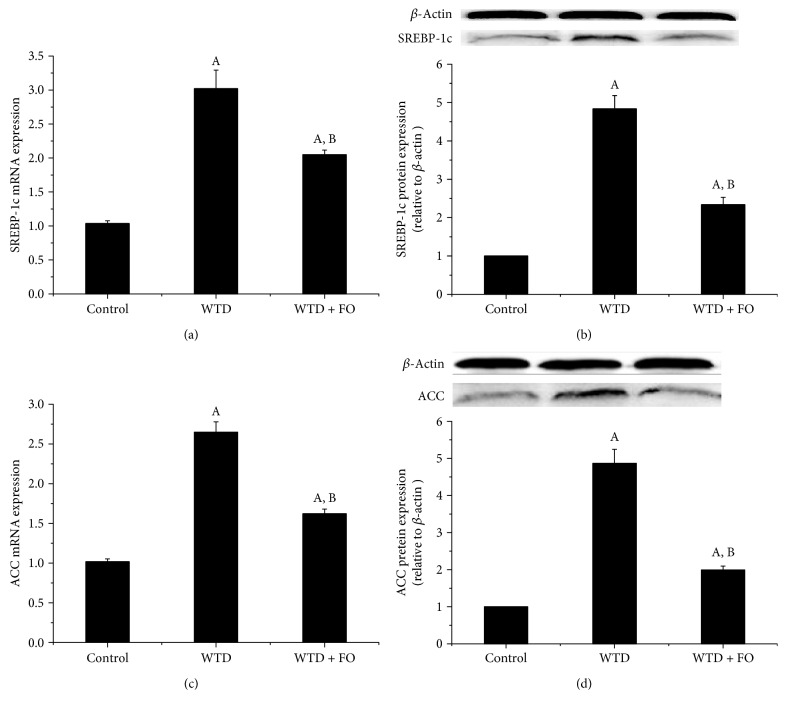
Flaxseed oil intervention decreased mRNA and protein expressions of hepatic SREBP-1c and ACC in mice. Quantitative real-time RT-PCR and Western blot analysis of the mRNA and protein expressions of (a-b) SREBP-1c and (c-d) ACC in the liver of mice in each group. Fold changes of mRNA levels were determined after normalization to internal control *β*-actin RNA levels. Blotting with anti-*β*-actin was used as a protein loading control. Protein expressions were presented as fold change relative to control. Representative immunoblots are shown. Each bar denotes mean ± SEM (*n* = 4 mice). ^A^*P* < 0.05 versus the control group; ^B^*P* < 0.05 versus the WTD group.

To investigate the underlying molecular mechanism by which dietary flaxseed oil modulates lipid metabolism, gene and protein expressions of the major factors involved in hepatic fatty acid catabolism and synthesis were detected. Results showed that flaxseed oil supplement apparently reversed the decreased mRNA and protein expressions of hepatic peroxisome proliferator-activated receptor alpha (PPAR*α*), carnitine palmitoyltransferase 1A (CPT1A), and acyl CoA oxidase 1 (ACOX1) induced by WTD (Figure 3). In addition, it was also observed that administrations of flaxseed oil significantly downregulated the mRNA and protein expressions of sterol regulatory element binding protein-1c (SREBP-1c) and acetyl-CoA carboxylase (ACC) (Figure 4).

### 3.4. Dietary Flaxseed Oil Inhibited Oxidative Stress

**Figure 5:**
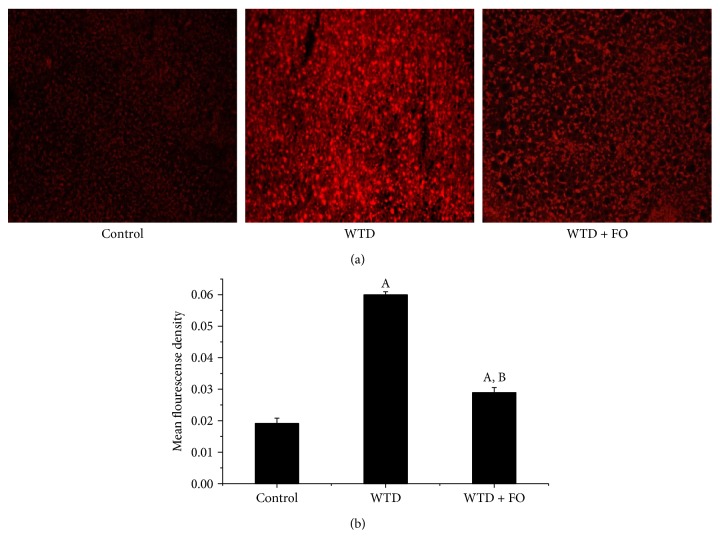
Effects of flaxseed oil intake on hepatic ROS production of mice. (a) ROS in the liver of the mice was detected by using DHE which reacts with ROS and forms ETH that binds to DNA and produces red fluorescence signal, visualized with a fluorescence microscope (**×**200) and quantified. (b) Fluorescence intensities in randomly selected areas of the images were quantified by using the IPP image analysis software. Data represent means **±** SEM and are normalized to % of field area. Each bar or point denotes mean ± SEM (*n* = 6 mice). ^A^*P* < 0.05 versus the control group; ^B^*P* < 0.05 versus the WTD group.

**Figure 6:**
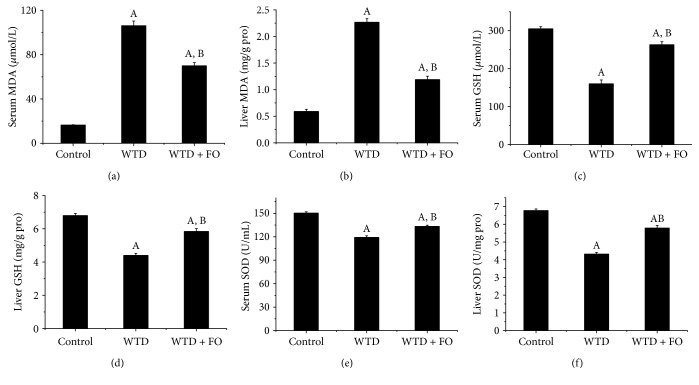
Effects of dietary flaxseed oil on the levels of MDA, GSH, and SOD in serum and liver of mice. (a) Serum MDA, (b) liver MDA, (c) serum GSH, (d) liver GSH, (e) serum SOD, and (f) liver SOD. Each bar or point denotes mean ± SEM (*n* = 12 mice). ^A^*P* < 0.05 versus the control group; ^B^*P* < 0.05 versus the WTD group.

In this experiment, in situ ROS production of liver was determined. As illustrated in Figures 5(a) and 5(b), high level of ROS in liver induced by WTD was significantly reduced after exposure to flaxseed oil. Moreover, flaxseed oil consumption apparently decreased the concentrations of MDA and elevated the levels of GSH and SOD in serum and liver (Figures 6(a), 6(b), 6(c), 6(d), 6(e), and 6(f)).

### 3.5. Intervention with Flaxseed Oil Decreased Hepatic Transaminase Activities

**Figure 7:**
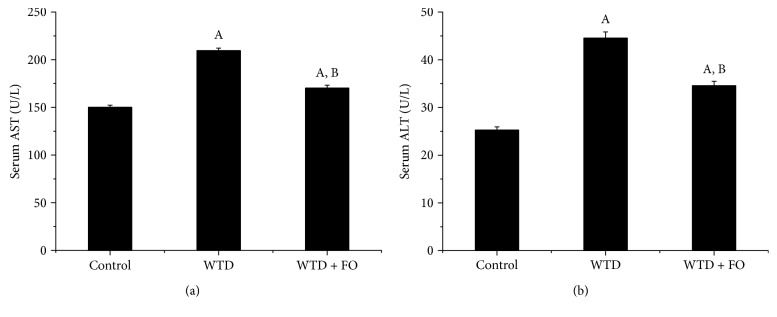
Flaxseed oil intake ameliorates serum transaminases in mice. (a) Serum AST. (b) Serum ALT. Each bar or point denotes mean ± SEM (*n* = 12 mice). ^A^*P* < 0.05 versus the control group; ^B^*P* < 0.05 versus the WTD group.

Plasma AST and ALT levels are important liver injury markers. Compared with the control group, the AST and ALT levels in mice fed WTD were significantly increased, whereas compared with the WTD group, the activity of AST and ALT in the WTD + FO group decreased by 22.4% (*P* < 0.05) and 18.7% (*P* < 0.05), respectively (Figures 7(a) and 7(b)).

### 3.6. Application of Flaxseed Oil Alleviated Inflammation in Mice

**Figure 8:**
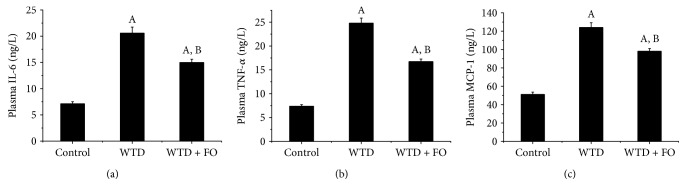
Dietary flaxseed oil alleviates plasma inflammatory cytokines in mice. (a) Plasma IL-6, (b) plasma TNF-*α*, and (c) plasma MCP-1. Each bar or point denotes mean ± SEM (*n* = 12 mice). ^A^*P* < 0.05 versus the control group; ^B^*P* < 0.05 versus the WTD group.

**Figure 9:**
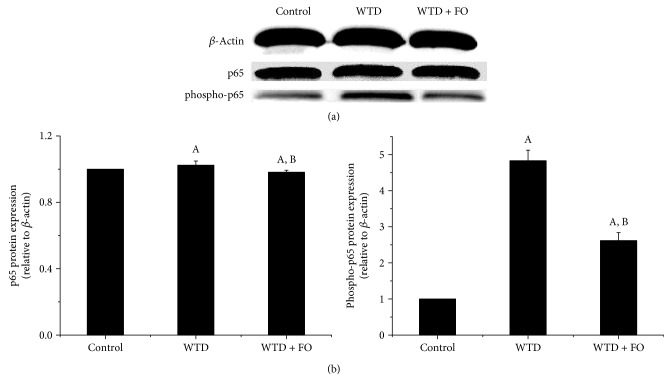
Effects of flaxseed oil intervention on hepatic protein expressions of phosphorylated p65 subunit of NF-*κ*B or total p65 in mice. Blotting with anti-*β*-actin was used as a protein loading control. Protein expressions were presented as fold change relative to control. Representative immunoblots are shown. Each bar denotes mean ± SEM (*n* = 4 mice). ^A^*P* < 0.05 versus the control group; ^B^*P* < 0.05 versus the WTD group.

**Figure 10:**
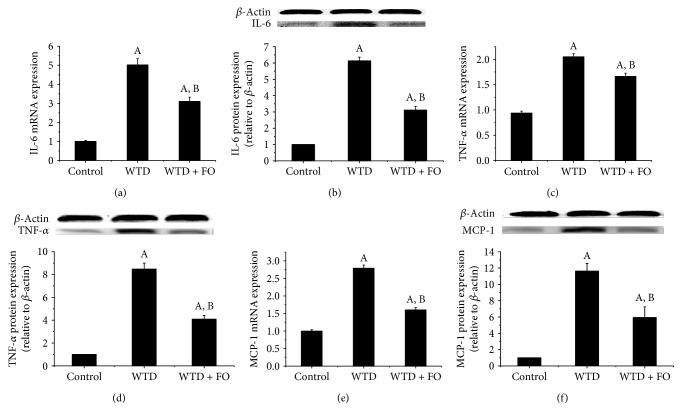
Supplementation of flaxseed oil decreased mRNA and protein expressions of hepatic inflammatory cytokines in mice. Quantitative real-time RT-PCR and Western blot analysis of the mRNA and protein expressions of (a) IL-6, (b) TNF-*α*, and (c) MCP-1 in the liver of mice in each group. Fold changes of mRNA levels were determined after normalization to internal control *β*-actin RNA levels. Blotting with anti-*β*-actin was used as a protein loading control. Protein expressions were presented as fold change relative to control. Representative immunoblots are shown. Each bar denotes mean ± SEM (*n* = 4 mice). ^A^*P* < 0.05 versus the control group; ^B^*P* < 0.05 versus the WTD group.

As illustrated in Figure 8, compared with control, mice fed on WTD exhibited significantly higher plasma levels of IL-6, TNF-*α*, and MCP-1. A significant reduction of IL-6, TNF-*α*, and MCP-1 was noted after flaxseed oil treatment. In an effort to seek an explanation for the inflammation induced by WTD and the protection effect exerted by flaxseed oil on this process, at the molecular level, we assessed the expression of nuclear factor-kappa B (NF-*κ*B), a crucial regulator of inflammation cytokines involved in NAFLD. The data clearly displayed that flaxseed oil-feeding mice had lower protein levels of phospho-p65, a transcriptionally active form of NF-*κ*B, compared with the mice given WTD (Figures 9(a) and 9(b)). In company with these results, the increased mRNA and protein expressions of NF-*κ*B target genes IL-6, TNF-*α*, and MCP-1 induced by WTD in liver were dramatically reversed by flaxseed oil intervention (Figures 10(a), 10(b), 10(c), 10(d), 10(e), and 10(f)).

## 4. Discussion

NAFLD is a burgeoning public health concern worldwide because of its high morbidity and its association with cardiovascular diseases and type 2 diabetes [22, 23]. In recent years, pathogenesis and treatment of NAFLD are attracting greater attention. A growing body of evidence emphasizes that increased serum levels of lipids as a result of WTD can cause fat accumulation, oxidative stress, and inflammation in the liver, which leads to NAFLD [24]. To simulate the pathogenesis of NAFLD as closely as possible to the human condition, we created a mouse model where NAFLD, lipid disorder, oxidative stress, and inflammation were induced by WTD in apoE-KO mice. This study provides the first evidence, to our knowledge, of dietary flaxseed oil-improved NAFLD induced by WTD via restoring impaired lipid metabolism, attenuating oxidative stress, and inhibiting inflammation.

Anomalous lipid metabolism plays a crucial role in the progression of NAFLD, attacking hepatocytes [25]. The latest study setup by Wang et al. demonstrated that dietary ALA-rich flaxseed oil prevented alcoholic hepatic steatosis via ameliorating lipid homeostasis at the adipose tissue-liver axis in mice [26]. Meanwhile, it has been well established that increased consumption of ALA was associated with improvements in the level of TG and gene expression involved in fatty acid metabolism [27, 28]. Consistent with previous research, in this work, we found that intervention of flaxseed oil decreased TG and TC in serum and liver, which indicated that supplement of flaxseed oil offered a strategy for optimizing lipid levels. To better explain the effects of flaxseed oil on the lipid, we further assayed mRNA and protein expressions of the factors in charge of hepatic lipid metabolism.

Excessive accumulation of TG in hepatocytes, as a precursor for NAFLD, is mainly composed of upregulated de novo fat synthesis and impaired fatty acid *β*-oxidation [29]. De novo fat synthesis in the liver converts excess carbohydrates into fatty acids and their esterified TG forms through the activity of many enzymes, including ACC, fatty acid synthase (FAS), and stearoyl-CoA desaturase 1 (SCD1). These lipogenic gene expressions can be regulated by a key transcriptional factor, SREBP-1c [30]. Devarshi et al. demonstrated that flaxseed oil diet improved lipid metabolism through downregulating SREBP-1c in diabetic rats [31]. Our study showed similar data with previous reports that flaxseed oil intervention decreased mRNA and protein expressions of SREBP-1c and ACC in liver. These results indicate that flaxseed oil supplementation may inhibit the SREBP-1c pathway to downregulate ACC expression, which in turn reduces TG synthesis.

On the other hand, it has been considered that n-3 PUFA exhibited TG-reducing effects through regulation of PPAR*α*, which controls hepatic fatty acid catabolism [32, 33]. PPAR*α*, which belongs to the super family of ligand activated nuclear hormone receptors, is thought to be the principal regulator in the fatty acid *β*-oxidation through modulation of CPT1A and ACOX1, the rate-limiting enzymes in mitochondrial and peroxisomal fatty acid oxidation, respectively [34]. In a previous study, flaxseed oil diet was observed to improve lipid metabolism through upregulating PPAR*α* in diabetic rats [31]. Recently, Rincón-Cervera et al. demonstrated that a larger supply of ALA modulated the fatty acid metabolism through increasing the expression and DNA-binding of PPAR*α* [35]. As we expected, in this work, administration of flaxseed oil upregulated mRNA and protein expressions of hepatic PPAR*α*, as well as its target genes CPT1A and ACOX1. This data made us speculate that increased *β*-oxidation of fatty acids might be responsible for the TG-lowering effect of flaxseed oil displayed in this study. Since the activations of PPAR*α* and SREBP-1c have been shown to stimulate fatty acid *β*-oxidation and TG synthesis, respectively, we believe that increased *β*-oxidation of fatty acid and reduced TG synthesis are likely responsible for the TG-lowering effect of flaxseed oil. Based on the important lipid modulation role of molecules mentioned above and the results in the present study, we suggest that improvement effects of flaxseed oil on lipid profiles and NAFLD may be linked to the regulation of SREBP-1c and PPAR*α*.

According to the classical “two-hit” hypothesis and modified “multiple-hit” hypothesis of NAFLD pathogenesis, oxidative stress is important in mediating the progression of NAFLD from steatosis to NASH, fibrosis, and cirrhosis [36]. An established source of oxidative stress is ROS generated by the free fatty acid metabolism in microsomes, peroxisomes, and mitochondria. ROS attack PUFA and initiate lipid peroxidation within cells, resulting in the formation of aldehyde by-products, such as MDA, which could activate the inflammatory response and, consequently, cause liver stellate cell fibrogenesis [37]. Recently, reports showed that dietary flaxseed oil was able to ameliorate renal oxidative stress in streptozotocin-nicotinamide-induced diabetic rats and modulate gamma irradiation and carbon tetrachloride-induced oxidative stress in the brain of female rats, which indicated that flaxseed oil treatment had an antioxidative function [38, 39]. In the present study, ROS production and the lipid peroxidation indicator MDA were increased in both serum and liver tissue in WDT-fed mice, whereas the levels of GSH and SOD, two potent antioxidants, were decreased in both serum and liver tissue. However, a pronounced reduction of ROS along with lower concentration of MDA and higher levels of GSH and SOD were observed in FO-fed animals than in mice on WTD. These results suggested that the potential effect of flaxseed oil in preventing oxidative stress could be due to the ability to reduce free radical production or through increased free radical scavenging activity. Antioxidant potential of flaxseed oil may represent another mechanism for alleviating and improving NAFLD.

Besides oxidative stress, inflammation is believed to be a substantial contribution that mediates “second-hits” to steatotic livers and exacerbates liver injury [40]. In the advanced stage of NAFLD, excessive fat acid accumulation in hepatocytes resulting from dysregulation of the lipid metabolism and oxidative stress will further activate inflammatory responses, which ultimately causes hepatic injury and fibrosis [41]. Consistent with this pathological progression of NAFLD/NASH, a growing body of evidence showed that the patients with NAFLD/NASH have elevated concentrations of IL-6, TNF-*α*, and MCP-1 [42, 43]. A recent study showed that dietary flaxseed oil ameliorated inflammation in streptozotocin-nicotinamide-induced diabetic rats [36]. In addition, Hendawi et al. demonstrated that flaxseed oil was able to protect against thiacloprid-induced hepatotoxicity [44]. Here, we found higher levels of IL-6, TNF-*α*, and MCP-1 accompanying elevated levels of AST and ALT in the WTD group than in the control, which indicated ongoing NASH and liver injury, while dietary flaxseed oil significantly reduced plasma levels of IL-6, TNF-*α*, and MCP-1 initially increased by WTD. Since these inflammatory markers have a key role in mediating inflammation, the positive impact of dietary flaxseed oil on IL-6, TNF-*α*, and MCP-1 in plasma observed in this research supported that beneficial function in NAFLD prevention of flaxseed oil is at least partly through perfecting inflammatory response. In order to investigate the reason for inflammation induced by WTD and the protection effect exerted by flaxseed oil on this process, at the molecular level, we assessed the expression of nuclear factor-kappa B (NF-*κ*B). NF-*κ*B is a nuclear protein factor that participates in the regulation of a variety of protein genes and causes disease through the induction of cytokines, which are related to immunity, inflammation, and fibrosis [45]. Several studies have found that the activation of NF-*κ*B could facilitate the occurrence and development of NAFLD, and the underlying mechanisms are related to inflammation and oxidative stress [46–48]. In addition, it has been published that NASH patients and rodent models of NASH had greater NF-*κ*B activation and protein expressions of NF-*κ*B-dependent proinflammatory genes including TNF-*α* and MCP-1 [49, 50]. Lately, an in vitro experiment conducted by Monk et al. showed that flaxseed oil cocultures reduced activation of inflammatory transcription factor NF-*κ*B (nuclear factor kappa-light-chain-enhancer of activated B cell) p65 in CD8(+) T cell [51]. This is corroborated in our study by flaxseed oil-mediated decrease in protein expression of phospho-p65, a transcriptionally active form of NF-*κ*B. The lower activity of NF-*κ*B and decreased mRNA and protein expressions of NF-*κ*B target genes IL-6, TNF-*α*, and MCP-1 in liver observed in the WTD + FO group indicate that the inhibition effect of flaxseed oil on inflammation may through regulating NF-*κ*B. In addition, since NF-*κ*B can be activated in a redox-dependent manner [52], we suggest that NF-*κ*B activation increase may be a result of oxidative stress induced by WTD and leads to inflammation which contributes to hepatic injury. Accordingly, the inhibition effect of flaxseed oil on NF-*κ*B described in this study may be also associated with its antioxidant activity.

## 5. Conclusion

The present study demonstrated that chronic WTD intake induced NAFLD. Dietary flaxseed oil compensated for WTD-induced lipid metabolism disorder, depressed hepatic oxidative stress, and inflammation, which contribute to the amelioration of NAFLD progress. The results suggest that flaxseed oil may be a potential dietary therapeutic tool against NAFLD.

## Figures and Tables

**Table 1:** Fatty acid composition of the experimental oils (%).

Fatty acid	Lard	Flaxseed oil
C14: 0	1.38	ND
C16: 0	29.29	5.79
C16: 1	2.20	ND
C18: 0	13.34	3.27
C18: 1	42.89	20.21
C18: 2	9.61	12.91
C18: 3	0.21	57.82
C20: 0	0.51	ND
C20: 1	0.57	ND
C22: 0	ND	ND

ND: not detected.

**Table 2:** Composition of the experimental diets (g/kg).

Components	Control group	WTD group	WTD + FO group
Cornstarch	397.5	321.3	321.3
Casein	200	200	200
Dextrinized cornstarch	132	106.7	106.7
Sucrose	100	80.8	80.8
Soybean oil	70	56.6	56.6
Fiber	50	40.4	40.4
Mineral mix	35	28.3	28.3
Vitamin mix	10	8.1	8.1
L-Cystine	3	2.4	2.4
Choline bitartrate	2.5	2	2
Lard	0	153.4	53.4
Flaxseed oil	0	0	100
Cholesterol	0	1.5	1.5
Energy (kcal/kg)	3042.3	4554.2	4554.2

**Table 3:** 

Gene	Forward primer	Reverse primer
PPAR*α*	5′-GGAGTGCAGCCTCAGCCAAGTT-3′	5′-AGGCCACAGAGCGCTAAGCTGT-3′
CPTIA	5′-AAGAACATCGTGAGTGGCGTC-3′	5′-AGCACCTTCAGCGAGTAGCG-3′
ACOX1	5′-GCCTTTGTTGTCCCTATCCGT-3′	5′-CTTCAGGTAGCCATTATCCATCTCT-3′
SREBP-1c	5′-TCCTTAACGTGGGCCTAGTCCGAAG-3′	5′-GCTCGAGTAACCCAGCACGGG-3′
ACC	5′-CGTTGGCCAAAACTCTGGAGCTA-3′	5′-CCCACATGGCCTGGCTTGGAG-3′
IL-6	5′-ATTTCCTCTGGTCTTCTGG-3′	5′-TGGTCTTGGTCCTTAGCC-3′
TNF-*α*	5′-TCTCATTCCTGCTTGTGG-3′	5′-ACTTGGTGGTTTGCTACG-3′
MCP-1	5′-GCAGGTGTCCCAAAGAA-3′	5′-GGTGGTTGTGGAAAAGG-3′

**Table 4:** Effect of flaxseed oil on body and liver weight in each group throughout the feeding period.

Parameters	Groups		
	Control	WTD	WTD + FO
Initial body weight (g)	13.08 ± 1.48	13.00 ± 1.08	13.05 ± 0.88
Final body weight (g)	29.26 ± 0.34	32.98 ± 2.11^a^	33.90 ± 1.81^a^
Body weight gain (g)	16.18 ± 0.31	19.98 ± 1.74^a^	20.85 ± 2.17^a^
Liver weight (g/100 g body weight)	3.82 ± 0.22	5.21 ± 0.36^a^	4.82 ± 0.36^a, b^

Values are given as means ± SEM (*n* = 12). ^a^*P* < 0.05 versus the control group; ^b^*P* < 0.05 versus the WTD group.
